# DNA Mismatch Repair and Oxidative DNA Damage: Implications for Cancer Biology and Treatment

**DOI:** 10.3390/cancers6031597

**Published:** 2014-08-05

**Authors:** Gemma Bridge, Sukaina Rashid, Sarah A. Martin

**Affiliations:** Centre for Molecular Oncology, Barts Cancer Institute, Queen Mary University of London, Charterhouse Square, London EC1M 6BQ, UK; E-Mails: g.bridge@qmul.ac.uk (G.B.); sukaina.rashid@qmul.ac.uk (S.R.)

**Keywords:** oxidative DNA damage, mismatch repair, ROS, chemotherapeutics, mitochondria

## Abstract

Many components of the cell, including lipids, proteins and both nuclear and mitochondrial DNA, are vulnerable to deleterious modifications caused by reactive oxygen species. If not repaired, oxidative DNA damage can lead to disease-causing mutations, such as in cancer. Base excision repair and nucleotide excision repair are the two DNA repair pathways believed to orchestrate the removal of oxidative lesions. However, recent findings suggest that the mismatch repair pathway may also be important for the response to oxidative DNA damage. This is particularly relevant in cancer where mismatch repair genes are frequently mutated or epigenetically silenced. In this review we explore how the regulation of oxidative DNA damage by mismatch repair proteins may impact on carcinogenesis. We discuss recent studies that identify potential new treatments for mismatch repair deficient tumours, which exploit this non-canonical role of mismatch repair using synthetic lethal targeting.

## 1. Introduction

Cellular macromolecules, such as lipids, proteins and nucleic acids, are constantly exposed to a barrage of potentially damaging reactive oxygen species (ROS). These include free radicals with unpaired electrons, such as the superoxide anion (O_2_^•−^), the hydroxyl radical (^•^OH), and non-radical hydrogen peroxide (H_2_O_2_). The majority of ROS found in aerobic cells are generated during normal cellular metabolism [1]. ATP, the intracellular means of energy transfer, is regenerated in the mitochondria through oxidative phosphorylation. Electrons from NADH and FADH_2_ are moved along the electron transport chain through a series of protein complexes, generating the proton gradient and resulting in the reduction of oxygen to form water. However, this is an inherently leaky chain and consequently electrons are continuously released in the form of O_2_^•−^, predominantly from the oxidative phosphorylation complexes I and III. In addition, inflammation can be a source of endogenous ROS [2]. ROS may also be generated by exogenous agents such as UV exposure, ionising radiation, carcinogenic compounds and redox-cycling drugs [3].

Much of the O_2_^•−^ released by endogenous and exogenous means is converted into H_2_O_2_ by superoxide dismutases (SOD) and superoxide reductases, but a proportion may react with nitric oxide to form peroxynitrite (ONOO-), a very strong oxidant [4]. H_2_O_2_ is highly diffusible through different cellular compartments but possesses low chemical reactivity and therefore is only directly responsible for modifying proteins via thiol groups [4]. In the context of DNA damage, ^•^OH is the most relevant oxidant species as it reacts with both purine and pyrimidine bases and the sugar moiety of the DNA backbone [1]. Hydroxyl radicals are formed in the presence of copper or iron catalysts from H_2_O_2_ and O_2_^•−^ via the Haber-Weiss and Fenton reactions [3]. These highly reactive radicals are particularly damaging as there are no known enzymes or biological binding partners that can neutralise their activity.

The carbon-carbon double bonds of DNA bases are sites of “attack” for ^•^OH due to the high electron density [1]. Addition reactions occurring at these sites generate C4-OH-, C5-OH- and C8-OH-adduct radicals of guanine and adenine and C5-OH- and C6-OH-adduct radicals of thymidine and cytosine [1]. Thymidine is also susceptible to the formation of an allyl radical when removal of a hydride anion (H^•^) from its methyl group occurs. These radical intermediates are then subjected to further oxidation and reduction reactions to generate a plethora of DNA lesions, including the major purine derivatives, 8-oxoguanine (8-oxoG) and 2,6-diamino-4-hydroxy-5-formamido-pyrimidine (FapyGua), and the oxidised pyrimidines cytosine glycol and thymine glycol (Table 1). 8-oxoG is the most stable of these lesions and can pair with both the original cytosine and also adenine during DNA replication [5]. If misincorporation of adenine across from 8-oxoG is allowed to persist, G:C to T:A transversions may occur. C to T transitions are another commonly observed mutation generated by oxidative damage, particularly due to the cytosine-derived products uracil glycol and 5-hydroxyuracil mispairing with adenine [1]. In fact, C to T transitions are the most frequent mutations found in human tumours and in the tumour suppressor gene *TP53* [6,7]. However, it must be noted that a significant proportion of C to T transitions may be attributed to a subclass of APOBEC cytidine deaminases that catalyse the irreversible hydrolytic deamination of cytidine to uridine, which is converted to thymidine upon subsequent DNA replication events [8].

**Table 1:** Outline of single oxidative DNA lesions and the DNA repair pathways responsible for their removal.

Original DNA Base	Oxidatively Induced Product	Repaired by	Proteins Implicated
Guanine 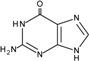	8-Oxoguanine 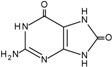	BER	OGG1; MUTYH
		MMR	MSH2; MSH6; MLH1
		NER	Multiple members
		dNTP pool sanitisation	MTH1
	2,6-Diamino-4-hydroxy-5-formamidopyrimidine 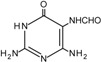	BER	OGG1; NEIL1; NEIL3
	Spiroiminodihydantoin 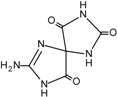	BER	NEIL1; NEIL3
	Guanidinohydantoin 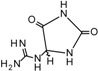	BER	NEIL1; NEIL3
Cytosine 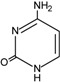	5-Hydroxycytosine 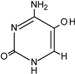	BER	NEIL2; NEIL3; NTH1
	5,6-Dihydroxycytosine 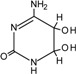	BER	NTH1
	5-Hydroxyuracil 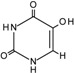	BER	NEIL2; NEIL3; NTH1
	5,6-Dihydrouracil 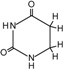	BER	NEIL2
Adenine 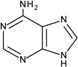	8-Hydroxyadenine 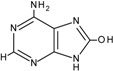	dNTP pool sanitisation	MTH1
	4,6-Diamino-5-formamidopyrimidine 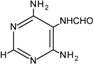	BER	NEIL1; NEIL3
	2-Hydroxyadenine 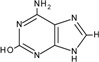	BER	MUTYH
		MMR	MSH2; MSH6
		dNTP poolsanitisation	MTH1
Thymine 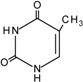	5-Hydroxy-6-hydrothymine 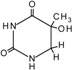	BER	NTH1
	Thymine glycol 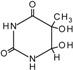	BER	NEIL1; NEIL3; NTH1
		MMR	MSH2
		NER	Multiple proteins
	5-Hydroxy-5-methylhydantoin 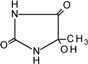	BER	NEIL1; NEIL3

Given the high rates of oxidative DNA damage (approximately 10^5^ lesions per cell per day) it is unsurprising that this source of DNA alteration has been discussed in relation to the aetiology of cancer. It has been reported that chronic oxidative stress, particularly in the context of chronic inflammation, is associated with carcinogenesis. For example, the connection between ulcerative colitis and colorectal cancer is well established and studies suggest that inflammatory mediators associated with this disease are responsible for the production of ROS, resulting in oxidative DNA damage and ultimately fixed alterations in cancer causing genes [9,10]. Numerous studies assessing DNA oxidative damage in malignant cells or tissues compared to non-malignant controls have identified higher levels of 8-oxoG and other markers of oxidative stress in the cancerous samples. This has been observed in breast, colorectal, gastric and lung cancer, among many others [11]. These observations could simply reflect a reduction in the ROS scavenging and DNA repair capabilities of the cell during carcinogenesis, rather than an actual cause of the disease. However; increasing evidence suggests that in some cases oxidative stress may be an initiating factor in the development of cancer [12].

## 2. DNA Repair of Oxidative DNA Damage

Due to the mutagenic nature of oxidatively-induced DNA lesions, several DNA repair mechanisms have evolved to remove them. Base excision repair (BER) and nucleotide excision repair (NER) both identify and excise DNA lesions before instigating polymerase and ligase activity to fill in the resultant gap; however they differ in the size of the lesions that they remove. The DNA glycosylases of BER, including uracil-DNA glycosylase (UNG), 8-oxoguanine DNA glycosylase (OGG1), nth endonuclease III-like 1 (NTHL1) and nei endonuclease VIII-like 1, 2 or 3 (NEIL1/NEIL2/NEIL3), all recognise different single base lesions (Table 1). They do this by gently pinching the DNA as they scan along it, causing the DNA to bend at the site of a damaged base, which flips out of the double helix and enters the binding site of the enzyme [13]. The DNA glycosylase then cleaves the N-glycosidic bond between the damaged base and the 2'-deoxyribose, generating an apurinic- or apyrimidinic-(AP) site. AP-sites must be further processed by an endonuclease, most commonly APE1, to create the correct 3' and 5' ends that allow DNA polymerase beta (POLB) and DNA ligase I (LIG1) to insert and ligate the nascent base [13].

Whilst BER acts to repair individual nucleotides, the NER system generally removes oligodeoxynucleotide chains containing large, DNA-distorting lesions such as intra-strand crosslinks, tandem lesions and bulky adducts [1]. This occurs either during normal surveillance of the genome or specifically on transcribing DNA strands. The damage-sensing proteins, DNA-damage binding (DDB) and XPC/RAD23B highlight sites of DNA damage on both transcribed and untranscribed DNA strands whilst the damage recognition signal in transcription-based NER occurs when RNA polymerase is stalled by a DNA lesion. Once the section of damaged DNA has been identified a complex containing XPA, ERCC1, XPF, TFIIH, XPG and RPA acts as an excinuclease to make two incisions in the DNA strand either side of the lesion. Similarly to BER, repair is completed by polymerases (δ, ε and/or κ) and ligases (LIG1 or LIG3/XRCC1) which fill and seal the resulting gap [14]. The only single nucleotide oxidatively-induced lesions that NER is known to repair are thymine glycol [15], 8-oxoG [16] and 8,5'-cyclopurine-2'-deoxynucleosides [17,18], the latter of which cannot be repaired by BER due to the 8,5'-covalent bond. The single base lesions repaired by BER and NER are highlighted in Table 1.

Although BER and NER have long been considered the main systems involved in combating ROS-induced injury to DNA, the mismatch repair (MMR) pathway has been implicated in the response to oxidative DNA damage, particularly 8-oxoG residues [19]. This aspect of MMR function and how it impacts on the development of potential new cancer therapeutics will be the focus of our review. We discuss the clinical implications of inducing oxidative DNA damage in cancer therapeutics and the emerging evidence suggesting a role for MMR in the repair of mitochondrial oxidative DNA damage.

## 3. The DNA Mismatch Repair Pathway

The primary role of the MMR pathway is the repair of base-base mismatches and insertion/deletion loops (IDLs). The key protein complexes instrumental to the functioning of the MMR system are the MutS and MutL families which are highly conserved from lower organisms to eukaryotes [20]. There are two heterodimeric complexes composed of MutS homologues (MSH): MutSα (comprised of MSH2 and MSH6) and MutSβ (comprised of MSH2 and MSH3). The key role of MutSα is the recognition of base-base mismatches and small IDLs. The sensing of longer IDLs is carried out by MutSβ and there is experimental evidence to suggest that MutSβ is unable to repair base-base mismatches but is able to overlap with MutSα in the repair of small IDLs [21,22].

Several heterodimers of MutL homologues (MLH) have also been identified; these include MutLα (complex of MLH1 and PMS2), MutLβ (complex of MLH1 and PMS1) and MutLγ (complex of MLH1 and MLH3). Evidence thus far suggests that the role of MutLα is to interact with MutS to orchestrate the recruitment of downstream repair proteins by signalling the recognition of a mismatch. However, information about the exact nature of these protein interactions and how they carry out their role is lacking. MutLγ is thought to be involved in repairing some IDLs as well as being involved in meiotic recombination [23,24,25,26].

A simplified overview of the MMR process is that MutS recognizes a DNA replication error and subsequently allows the recruitment of MutL protein complexes which coordinate downstream proteins necessary to finalise DNA repair. The MMR system is able to access both the mismatch on the DNA and the strand discrimination signal which is a strand-specific nick allowing discrimination between daughter and template DNA. There are several proposed mechanisms of the MMR system, which fall under the two main headings of “moving models” and “stationary models”. The moving models essentially result in the MutS/MutL complexes leaving the mismatch they have encountered, made possible by the fact that these MMR complexes possess ATPase activity. The resulting moving clamps diffuse along the DNA in a uni (“translocation model”) or bi-directional manner (“sliding clamp model”) from the mismatch. Repair of these lesions takes place when one of these clamps comes across a strand break that is associated with the accessory proteins proliferating cell nuclear antigen (PCNA) and replication factor C (RFC). The “stationary” model, which is also termed the “DNA bending/verification” model, proposes that the MMR complexes remain at the mismatch allowing the DNA to bend or loop, thereby permitting contact between the mismatch and the strand discrimination signal. The other key proteins involved in the MMR system are exonucleases, such as exonuclease 1 (EXO1), which remove the error from the daughter strand; DNA polymerases, which are required for DNA synthesis; and finally DNA ligase 1 (LIG1), which joins up the gaps in the DNA sequence [20,27].

## 4. The Role of MMR in Oxidative Damage Repair

There is mounting evidence that the DNA MMR pathway has several other non-canonical roles including participating in homologous recombination, mitotic and meotic recombination, repair of double strand breaks, immunoglobin class switching and coactivation of oestrogen receptor alpha [24,28]. Emerging evidence suggests that the MMR system is involved in the response to oxidative DNA damage and this is possibly linked to carcinogenesis due to an accumulation of oxidative DNA damage in the context of MMR deficiency [29,30].

MMR deficiency has been observed in 15%–17% of all primary colorectal cancer (CRC) [31,32], 30% of endometrial cancers [33] and approximately 10% of ovarian cancers [34]. Lack of one or more MMR genes in these cancers has been attributed to epigenetic silencing, in addition to both germline (inherited) and somatic (acquired) mutations [34,35,36]. Lynch syndrome, an autosomal dominant condition associated with a predisposition to several cancers, occurs due to inheritance of a single amorphic mutation in *MSH2*, *MSH6*, *MLH1* or *PMS2*, followed by subsequent sporadic loss of the second allele [37]. In cells lacking an effective MMR pathway mutations are left unrepaired and can build up in the genome, leading to a “mutator phenotype”. Therefore MMR-deficient tumour cells possess mutation rates that are 100–1000-fold greater than that of normal cells [38,39]. The mutator phenotype leads to downstream mutations in tumour suppressor genes and oncogenes, particularly in those harbouring microsatellites such as *PTEN*, *BAX*, *IGF2R* and *TGFBR2*, thereby promoting tumourigenesis [35,36,40]. Upon loss of MMR, microsatellite sequences can become unstable and consequently shorten or lengthen, a phenomenon termed microsatellite instability (MSI) [34].

Numerous studies report that loss of key MMR genes decreases the efficiency of repair of DNA lesions caused by oxidative damage. Embryonic stem (ES) cells and mouse embryo fibroblasts (MEFs) derived from mice deficient in MSH2 (*Msh2*^+/−^ and *Msh2*^−/−^) have higher levels of both basal and ROS-induced genomic 8-oxoG in comparison to wild-type controls [41,42]. It was initially shown that MSH2-deficient cells had more oxidative damage (8-oxoG and thymine glycol) when exposed to low-levels of ionising radiation [42]. A subsequent study by Colussi* et al*. showed that baseline 8-oxoG levels were higher in DNA extracted from MSH2 and MLH1 deficient cell lines [41]. This was attributed to impaired removal of 8-oxoG that had been incorporated into DNA from the oxidised dNTP pool. Overexpression of MTH1, the hydrolase that removes 8-oxodGTP, reduced genomic 8-oxoG levels to a greater extent in MMR-deficient* versus* MMR-proficient mouse CRC cells and was reported to reduce the mutator phenotype in *Msh2*^−/−^ MEFS [41]. This finding was supported by an earlier study, which also showed that 8-oxoG levels were more responsive to exogenous MTH1 in *Msh2*^−/−^ cells [43]. Expression of the *mutS* homologue of *Helicobacter pylori* (*H. pylori*) has been shown to reduce oxidative DNA damage [44]. In response to *H. pylori* infection, the gastric mucosa undergoes an inflammatory oxidative stress response resulting in increased ROS levels. *mutS* mutant variants were more sensitive to ROS inducing drugs including H_2_O_2_ and had increased 8-oxoG accumulation when exposed to oxidative stress, in comparison to wild type *mutS* [44]. Mazurek* et al*. further elucidated the distinct roles of the MMR proteins in oxidative repair by showing that MutSα, but not MutSβ is activated by the presence of a mismatched 8-oxoG lesion [45]. Furthermore, MutSα was able to bind different mismatches with differing affinities: 8-oxoG/T>8-oxoG/G>8-oxoG/A>8-oxoG/C≈G/C. Because MutSα is only activated by mismatched 8-oxoG, the MMR system continually excises and resynthesizes the DNA base opposite an 8-oxoG lesion until a C is inserted opposite the lesion, forming a substrate that MutSα does not recognise. This ultimately results in a DNA substrate that can be excised by OGG1. The authors note that their findings are at odds with the conclusions of Ni* et al*., but that the observed differences may be due to differences between the mammalian and yeast systems [45,46].

Clustered DNA lesions are well recognised to be one of the consequences of DNA damage following exposure to chronic oxidative stress. They occur as a consequence of low energy electrons resulting in two or more DNA lesions, within one to ten base pairs. The DNA lesions can arise on a single strand or are bistranded. Bistranded lesions are further classified into double-strand break (DSB) or non-DSB (single strand breaks (SSBs); oxidative base damage; AP sites) clusters. Non-DSB clusters mostly contain oxidized base damage and are also known as bistranded oxidatively induced clustered lesions (OCDLs) [11,47]. The repair of oxidized base damage within clusters is partially performed by BER enzymes but the resulting lesions are thought to be highly relevant in cancer biology since they have a high mutagenic potential and are difficult to repair. Holt *et al*. have shown that the MSH2-deficient cells were less able to repair all lesions caused by ionizing radiation, including OCDLs, compared to the MSH2-proficient cells [48]. This role of MSH2 in the repair of oxidative damage within clustered DNA lesions was further investigated by Zlatanou* et al*. [49]. DNA polymerase Polŋ was found to interact with the H_2_O_2_–induced monoubiquitinated form of PCNA (mUb-PCNA) and this process was reduced in MSH2-deficient cells. Furthermore, the presence of MSH2-MSH6 was required for this modification of PCNA and recruitment of Polη to chromatin, and the ultimate removal of OCDLs [49]. In addition to 8-oxoG, thymine glycol and OCDLs, MMR has also been implicated in the removal of 2-hydroxyadenine in the context of frameshifts. MutSα was shown to recognise 2-hydroxyadenine-containing structures that resembled slipped-mispaired intermediates [50]. The authors hypothesised that MMR may therefore act to repair frameshifts caused by oxidation of adenine within repetitive sequences.

### How the Labour Is Shared: BER vs. MMR

**Figure 1:**
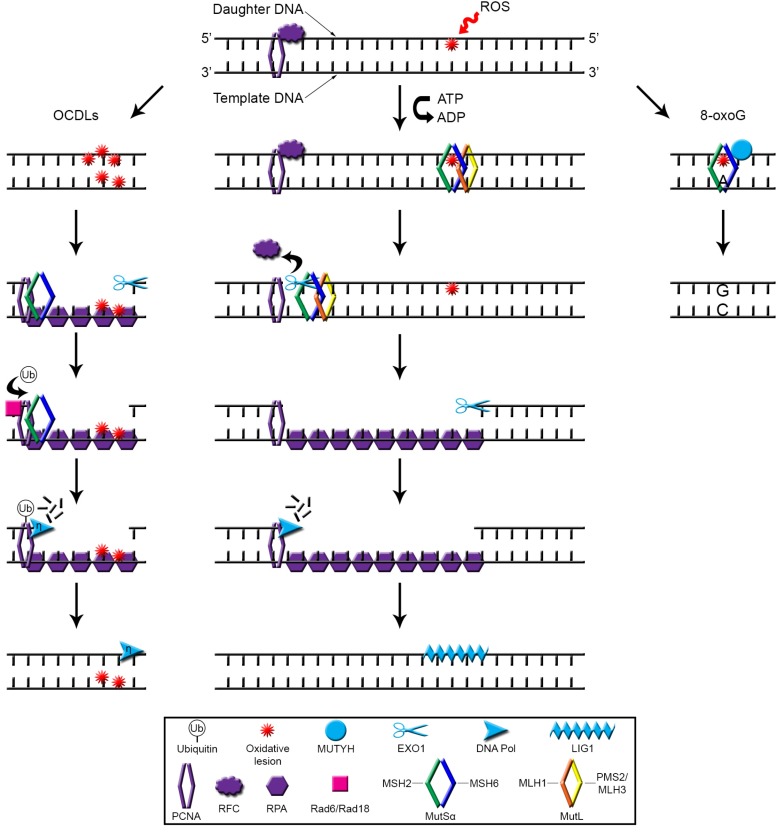
Repair of oxidative DNA lesions by the MMR pathway. Schematic representation of the hypothesised mechanisms of oxidative damage removal by MMR. ROS can induce lesions in the DNA, such as oxidation of guanine to 8-oxoG. These are recognised by MutSα which recruits MutL. The sliding clamp model of MMR proposes that the ternary MutSα/MutL complex moves along the DNA until it encounters RFC and PCNA bound to the 5' end of the daughter strand. RFC is consequently displaced which allows the recruitment EXO1 that mediates the degradation of the region containing the oxidative lesion. The resulting single-stranded gap is stabilised by the presence of RPA. Once the oxidative lesion is removed, EXO1 activity is no longer promoted by MutSα and is actually inhibited by MutL. A DNA polymerase (DNA Pol) synthesises new DNA to fill the gap and this is connected to the daughter strand by LIG1. The BER DNA glycosylase MUTYH has been shown to bind to MutSα via MSH6 whilst MutSα enhances the removal of 8-oxoG/A mismatched lesions by MUTYH. Tandem lesions of 8-oxoG and 5-hydroxyuracil within OCDLs may be recognised by MutSα, leading to the recruitment of EXO1 and removal of all or part of the lesion. PCNA is then loaded onto the resulting 3'OH ssDNA gap, possibly due to a direct interaction with MutSα. Rad6/Rad18 mediates the monoubiquitination of PCNA and MutSα is required for this process. Monoubiquitinated PCNA recruits DNA Polη, which synthesises new DNA across the damaged or undamaged template.

Studies in yeast by Ni* et al*. analysing MMR and BER contributions to oxidative DNA damage repair, identified increased mutation rates due to G:C-to-T:A tranversions in combined MMR and BER mutants (*MSH2*/*MSH6*+*ogg1*) compared to the single *ogg1* mutants [46]. Increased oxidative DNA damage upon MMR deficiency has been demonstrated in* in vivo* models as increased 8-oxoG levels were detected in the spleen, liver, heart, lungs, kidneys and small intestine of *Msh2*^−/−^ mice [30]. Interestingly, a synergistic accumulation of 8-oxoG has been described in several organs of *Msh2*^−/−^/*Mutyh*^−/−^ animals [51]. MutY homolog (MUTYH) is a DNA glycosylase of the BER pathway that removes 8-oxoG, therefore this data suggests that both MSH2 and MUTYH make a significant contribution to the repair of oxidative DNA damage. Interestingly, Gu* et al.* have shown that MUTYH physically associates with MutSα via MSH6 and that the removal of 8-oxoG/A mismatched lesions by MUTYH is enhanced by MutSα* in vitro* [52]. The authors propose that protein-protein interactions may be a means by which BER and MMR cooperate to reduce replicative errors caused by oxidative damage [52]. A summary of the evidence to date on how the MMR system potentially operates to repair oxidative DNA damage is shown in Figure 1.

## 5. Repair of Oxidative Damage to the Mitochondrial Genome

Mitochondrial DNA (mtDNA) is particularly prone to oxidative DNA damage for a variety of reasons, including its close proximity to the electron transport chain, where the majority of ROS is generated, and the fact that it is not protected by histones [53]. It has been established that mitochondria utilise BER as their primary mechanism for repairing mitochondrial oxidative DNA damage [54]. Nevertheless, there is increasing evidence that some form of MMR machinery is present in the mitochondria and that MMR proteins are potentially also involved in the repair of oxidative DNA damage to mtDNA. The first suggestion that an element of the MMR pathway may be involved in the repair of mtDNA came from reports that identified the presence of MSI in mtDNA of human CRCs. Habano* et al*. examined nine microsatellite sequences in the mtDNA of 45 sporadic CRCs and found that in 44% of these cancers there was an alteration in a polycytidine (C)n tract within the non-coding displacement-loop (D-loop) region and that three of the samples exhibited frameshift mutations within microsatellite tracts in NADH dehydrogenase genes [55]. 

The precise details of the MMR pathway has not been fully elucidated in mammalian mitochondria but a mitochondrial MutS homolog (Msh1) has been identified in *Saccharomyces cerevisiae*. Furthermore, Hashiguchi* et al*. identified mismatch-binding activity in mitochondrial extracts of three different human cell lines [56]. The authors predicted, using polyacrylamide gel migration, that the protein involved is 80–90 kDa and also binds to oxidative DNA lesions. Mismatch binding activity in mitochondrial extracts was also seen in MSH2-deficient cells, suggesting that mismatch binding in the mitochondria is MSH2 independent [57,58]. DeSouza* et al*. proposed that the key proteins involved in the human mitochondrial MMR system, are likely to be distinct from those of the conventional nuclear MMR pathway, as they could not detect mitochondrial localisation of MLH1, MSH3 and MSH6 in human HeLa cells by immunofluorescence [58]. However, they identified the repair factor Y-box binding protein (YB-1) as required for mitochondrial MMR, such that silencing of *YB-1* reduced MMR activity in mitochondrial extracts [58].

**Figure 2:**
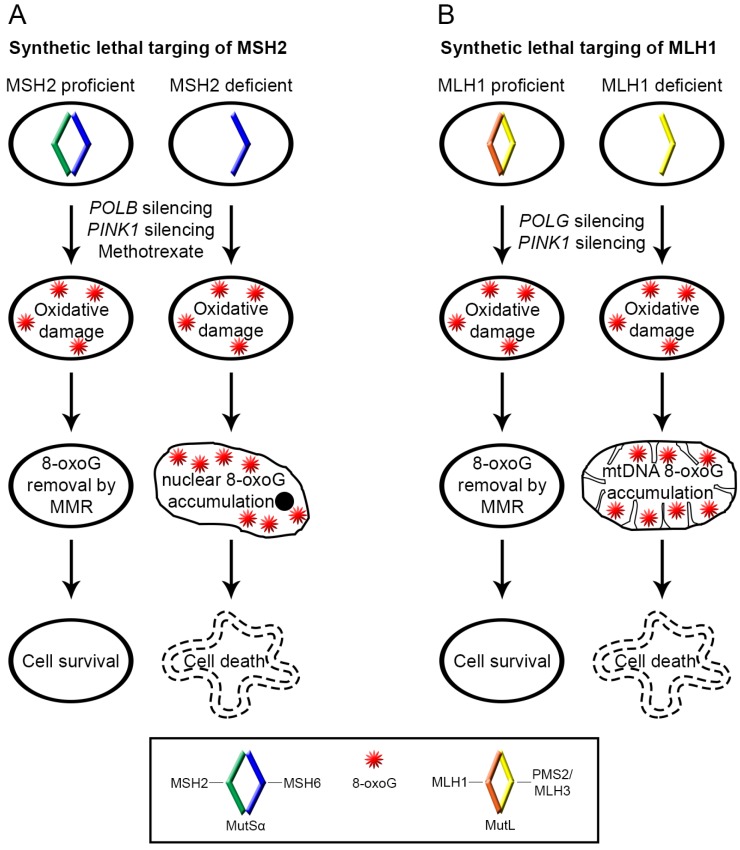
Accumulation of oxidative DNA damage causes synthetic lethality in MSH2 and MLH1 deficient cells. Silencing of *POLB*, *POLG* and *PINK1* or treatment of cells with the chemotherapeutic drug, methotrexate causes 8-oxoG lesions in DNA which can be successfully repaired in MMR competent cells. However, in MSH2 (**A**) or MLH1 (**B**) deficient cells 8-oxoG lesions accumulate in the nuclear (**A**) and mitochondrial (**B**) DNA respectively, leading to cell death.

We have recently shown further evidence of a potential role for MMR in the mitochondria, specifically requiring MLH1 but not MSH2. Our data suggest that silencing of *POLB* is synthetically lethal with MSH2 deficiency due to an increase in nuclear 8-oxoG lesions (Figure 2a) [59]. Whereas, silencing of the mitochondrial DNA polymerase γ (*POLG*) is synthetically lethal with MLH1, but not MSH2 deficiency, due to an increase in mitochondrial 8-oxoG lesions (Figure 2b) [59]. We also observed a small fraction of the MLH1 protein pool localized to the mitochondria, validated by studies in the mitochondrial proteome from mouse liver extracts [60]. Furthermore, we have shown that silencing of PTEN-induced putative kinase (*PINK1*) is synthetically lethal with MLH1, MSH2 and MSH6 deficiency, due to an increase in both mitochondrial and nuclear 8-oxoG lesions [61]. Taken together, these studies suggest a possible role for MLH1 in the repair of oxidative mtDNA damage, which needs to be further investigated.

## 6. MMR and Oxidative Damage: Relevance to Carcinogenesis

Given the dual function of MMR in removing both incorrectly placed bases and oxidatively damaged nucleotides, it is inherently difficult to dissect the separate impact of these two roles upon cancer development. The mutator phenotype is clearly the driving force behind carcinogenesis in many MMR-deficient tumours but does reduced 8-oxoG removal also contribute, either via increased MSI or independently? 

Few studies have examined the specific role of oxidative damage repair by the MMR pathway in relation to tumourigenesis. Colussi* et al*. tested to what extent oxidative DNA damage played a role in the MMR mutator phenotype by expressing MTH1 in MSH2-deficient MEFs; the resulting decrease in DNA 8-oxoG levels translated into a decrease in the mutator effect [41]. Glaab *et al*. reported that growing the MLH1 deficient CRC cell line HCT116 in the antioxidant ascorbate, both with and without H_2_O_2_ treatment, significantly reduced mutation rates and reduced MSI by 30% [62]. Conversely, it has been suggested that MLH1 deficient, HCT116 cells are less sensitive to H_2_O_2_ than their MMR-proficient counterparts (HCT116+Chr3) [62,63]. This was attributed to an impaired apoptotic response in the HCT116 cells, suggesting that MMR is required for the recognition of severe oxidative damage and subsequent signalling to the apoptotic machinery. Our data suggest that MSH2-deficient cells are more sensitive to treatment with H_2_O_2_ [64]. It has recently been shown in a model for oxidative damage-induced tumours that loss of MSH2 significantly increased the formation of epithelial tumours in the small intestine [65]. Upon treatment with potassium bromate, *Msh2*^−/−^ mice displayed a 22.5-fold increase in tumour incidence [35,65].

The evidence thus far suggests that the MMR system may supresses carcinogenesis in the context of oxidative damage by directly repairing ROS induced DNA lesions or acting as a sensor of oxidative damage, thereby activating apoptosis.

## 7. Therapeutic Targeting of MMR-Deficient Tumours with Oxidative Damage

Clinically, it would seem logical to use anti-oxidant or ROS scavenging agents in the treatment of cancers since oxidative DNA damage has been shown to induce mutagenesis. However, several large studies testing a variety of drugs (β-carotene, vitamin E, vitamin C, selenium, retinol, zinc, riboflavin and molybdenum) have not been able to definitively confirm that this approach is effective [66]. This area of research is still on-going and several clinical trials involving antioxidants in the treatment of cancer are currently recruiting patients. One such trial is a randomized placebo controlled intervention and follow-up trial examining the effects of the antioxidant vitamin B-6, homocysteine, oxidative stress and DNA methylation in patients with colorectal cancer compared to those with colorectal polyps (NCT01426490). A similar phase 2/3 study aims to establish the effect of vitamin B-6 and coenzyme Q10 on oxidative stress and antioxidant capabilities in hepatocellular carcinoma (NCT01964001). There is also an on-going pilot study assessing the use of the anti-oxidant N-acetyl- cysteine in early stage breast cancer and its effects on cancer progression (NCT01878695).

Several conventionally used chemotherapeutic agents are known to increase ROS and oxidative stress in addition to their main mechanism of action, including anthracyclines, platinum agents, epipodophyllotoxins and camptothecins [67,68]. There has also been a recent interest in studying specific ROS inducing agents as single treatments or in combination with conventional chemotherapy. A recent* in vitro* study examining the ROS inducing agent parthenolide and its soluble analogue dimethylamino parthenolide in triple negative breast cancers has shown that these drugs activate NADPH oxidase leading to the production of superoxide anions, and depletion of glutathione and *in vivo* studies showed a significant reduction in tumour growth and increased survival [69]. The addition of methylseleninic acid (MeSe) to cisplatin can increase cytotoxicity and selectivity of tumour cells, due in part to oxidative stress [70].

The ROS inducing drug, β-Lapachone is currently being studied in a phase 2 clinical trial (NCT01502800). Pre-clinical studies suggest that β-Lapachone exerts its cytotoxic effect by undergoing futile redox cycles on the detoxifying enzyme NAD(P)H:quinone oxidoreductase (NQO1). NQO1 levels are known to be elevated in several solid tumours and in these cancers, β-Lapachone results in excessive ROS formation [71]. Several other ROS inducing agents have been tested in early phase clinical trials but there are currently no drugs that have reached large scale phase 3 trials, aside from conventionally used chemotherapies with ROS generating properties, [69,70]. An alternative approach for targeting cancers by increasing oxidative stress is the use inhibitors of the BER enzyme, PARP, which is involved in the repair of oxidative damage, in combination with chemotherapeutics such as carboplatin, which induce ROS [72].

We have previously shown that treatment with the drug methotrexate is selectively lethal with MSH2-deficient cell lines, due to an increased susceptibility to oxidative stress [64]. The levels of 8-hydroxyguanine (8-OHG), a precursor to 8-oxoG, were similar in the MSH2-proficient and deficient cell lines following methotrexate treatment, but over time 8-OHG levels returned to baseline in the MSH2-proficient cell lines. Significantly, levels of oxidative DNA damage remained elevated in the MSH2-deficient cell lines. Our data suggest that in the absence of MSH2, oxidative DNA damaging agents such as methotrexate can cause damage, which is not efficiently repaired and its accumulation results in loss of cell viability (Figure 2a) [64]. These findings have been translated into an on-going clinical trial in metastatic colorectal cancer (NCT00952016). It has also been shown that cytosine based nucleoside analogs are selectively lethal with MLH1 and MSH2-deficient cell lines due to an increase in ROS levels and elevated oxidative DNA damage leading to apoptosis [73]. Furthermore, we have emerging evidence to suggest that MLH1-deficient tumours are selectively sensitive to mitochondrial-targeted agents, which are known to induce ROS as one of their main modes of action. Recently, Dwyer *et al*. have shown that several conventionally used antibiotics produce ROS as part of their mechanism of action [74]. Upon MutS overexpression, *E. coli* were increasingly resistant to treatment with these ROS-inducing antibiotics [74]. The authors concluded that the post replicative repair function of MMR was likely to be responsible for this effect but other roles of MMR could not be ruled out. It is therefore feasible that the role of MMR in the repair of oxidative DNA damage could also be contributing to the observed increase in bacterial survival. It would be interesting to investigate whether MMR-deficient cells are synthetically lethal with the antibiotics used in this study and whether this is as a result of increased oxidative DNA damage.

Concerns have been raised regarding modulating ROS homeostasis to treat tumours. Although ROS can cause oxidative DNA damage, it is also involved in the regulation of molecules involved in tumour biology and therefore could play a role in inhibiting carcinogenesis. There are therefore worries that antioxidants could promote tumourigenesis [75]. Excessive ROS generation and oxidative stress can also interfere with cancer treatment, such that apoptosis can be inhibited by high levels of ROS. Additionally, ROS can deregulate cell cycle progression, thereby interfering with cell-cycle targeted drugs [68]. There are also concerns that excessive oxidative stress may result in increased tumourigenesis and unacceptable toxicities [61]. It is perhaps important to remember that several chemotherapies currently in clinical use induce ROS as part of their mechanism of action and they have been given safely with tolerable toxicities. Furthermore, newer targeted treatments should in theory minimize toxicity by exploiting synthetic lethal approaches to treating tumour cells, whilst not harming the normal cells.

## 8. Conclusions

Although there is certainly evidence suggesting a role for the MMR pathway in the repair of oxidative DNA damage, the precise nature of this pathway is far from being fully clarified. Despite this, we and others have utilised high throughput screens to identify several molecules, drugs and compounds which are synthetically lethal with MMR deficiency as a consequence of increased oxidative DNA damage. These approaches provide a step towards understanding the complex link between the MMR pathway, oxidative damage and cancer as well as identifying promising new treatments and drug targets for MMR-deficient tumours.
